# P-2360. Wastewater-Based Surveillance of Hepatitis A Virus Across Communities in Alberta, Canada

**DOI:** 10.1093/ofid/ofae631.2511

**Published:** 2025-01-29

**Authors:** Benson Weyant, Aito Ueno, Barbara Jean M Waddell, Jangwoo Lee, Kevin Xiang, Kristine Du, Aidan Bender, Gail Visser, Janine McCalder, Chloe Papparis, Maria Bautista Chavarriaga, Kevin Fonseca, Mark Swain, Carla Coffin, Bonita Lee, Xiao-Li Pang, Steven J Drews, Christine O’Grady, Casey R J Hubert, Michael Parkins

**Affiliations:** University of Calgary, Calgary, Alberta, Canada; University of Calgary, Calgary, Alberta, Canada; University of Calgary, Calgary, Alberta, Canada; University of Calgary, Calgary, Alberta, Canada; University of Calgary, Calgary, Alberta, Canada; University of Calgary, Calgary, Alberta, Canada; University of Calgary, Calgary, Alberta, Canada; University of Calgary, Calgary, Alberta, Canada; University of Calgary, Calgary, Alberta, Canada; University of Calgary, Calgary, Alberta, Canada; University of Calgary, Calgary, Alberta, Canada; University of Calgary, Calgary, Alberta, Canada; University of Calgary, Calgary, Alberta, Canada; University of Calgary, Calgary, Alberta, Canada; University of Alberta, Edmonton, Alberta, Canada; University of Alberta, Edmonton, Alberta, Canada; Canadian Blood Services, Edmonton, Alberta, Canada; University of Calgary, Calgary, Alberta, Canada; University of Calgary, Calgary, Alberta, Canada; University of Calgary, Calgary, Alberta, Canada

## Abstract

**Background:**

Hepatitis A virus (HAV) incident infection in Canada is rarely diagnosed (i.e. incidence of 3.6-10 cases/100,000 persons) (PMID: 18159360). Infections generally relate to imported contaminated food-products, or travelers returning from endemic countries. Due to its fecal-oral spread, possible underdiagnosis, and often-cryptic presentation, HAV is an ideal candidate for wastewater (WW)-based surveillance, a tool increasingly utilized to monitor infectious diseases globally.

**Figure 1.**
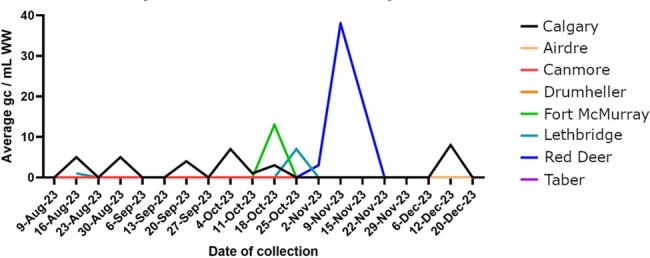
Longitudinal monitoring of HAV RNA wastewater abundance across eight Alberta municipalities over a 4-month period.

**Methods:**

24-hour composite WW was collected weekly from eight geographically disparate, and socioeconomically diverse municipal WW treatment plants in Alberta from August to December 2023. After short-term cold storage, WW was centrifuged, and RNA from the raw pellet was extracted using Qiagen’s RNeasy PowerFecal pro. HAV levels were quantified by RT-qPCR of the *vp1* gene. 2021 Canadian census data was used to define population demographics for each participating site.

**Figure:**
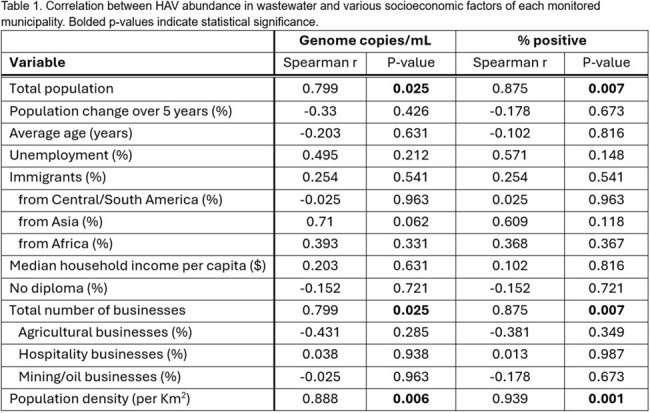

**Results:**

HAV was detected in 18/117 (15.4%) WW samples and 5/8 (62.5%) municipalities over the 4-month period (Figure 1). RNA abundance in HAV positive WW samples was a median of 3.4 copies/mL (IQR 0.44 - 6.97). Larger population size (p=0.007), and greater density (p=0.001) were associated with increased likelihood of HAV WW detection, whereas social and economic demographics of populations within sewershed catchments did not associate with likelihood of HAV detection (Table 1).

**Conclusion:**

HAV RNA is rarely detected in the wastewater of Alberta. Detection was more frequently observed in larger municipalities, which is consistent with non-endemic, imported disease. WW surveillance can potentially be adapted to monitor HAV in the context of outbreaks to reduce secondary transmission, and safeguard public health.

**Disclosures:**

Mark Swain, MD MSc, Abbott: Advisor/Consultant|Advanz: Advisor/Consultant|Gilead, BMS, CymaBay, Intercept, Genfit, Pfizer, Novartis, Astra Zeneca, GSK, Celgene, Novo Nordisk, Axcella Health Inc., Merck, Galectin Therapeutics: Grant/Research Support|GSK: Advisor/Consultant|Ipsen: Advisor/Consultant|Novo Nordisk: Advisor/Consultant Carla Coffin, MD MSc, Altimmune: Grant/Research Support|Gilead: Grant/Research Support|GSK: Grant/Research Support|Janssen: Grant/Research Support Steven J. Drews, PhD FCCM D(ABMM), Abbott: Grant/Research Support|Danaher: Honoraria|Roche: Advisor/Consultant|Roche: Grant/Research Support

